# Genetic diversity of *Contracaecum rudolphii* sp. A (Nematoda: Anisakidae) parasitizing the European Shag *Phalacrocorax aristotelis desmarestii* from the Spanish Mediterranean coast

**DOI:** 10.3389/fvets.2023.1122291

**Published:** 2023-02-02

**Authors:** Xavier Roca-Geronès, Roser Fisa, Isabel Montoliu, Margarida Casadevall, Carles Tobella, Josep M. Bas, Marialetizia Palomba, Simonetta Mattiucci

**Affiliations:** ^1^Secció de Parasitologia, Departament de Biologia, Sanitat i Medi Ambient, Facultat de Farmàcia i Ciències de l'Alimentació, Universitat de Barcelona, Barcelona, Spain; ^2^Section of Parasitology, Department of Public Health and Infectious Diseases, Sapienza-University of Rome, Rome, Italy; ^3^Department of Environmental Sciences, Faculty of Sciences, University of Girona, Girona, Spain; ^4^Department of Ecological and Biological Sciences, Tuscia University, Viterbo, Italy

**Keywords:** *Contracaecum rudolphii* sp. A, European Shag, Western Mediterranean, molecular identification, population genetics

## Abstract

Sibling species of the *Contracaecum rudolphii* (s.l.) complex are habitual endoparasites of cormorants of the Phalacrocoracidae family, worldwide. In Europe, the two species, *C. rudolphii* sp. A and *C. rudolphii* sp. B, have been identified. However, information regarding the occurrence and distribution of these anisakids in cormorants from Spain is scarce. In the present study, 20 specimens of the European Shag, *Ph. aristotelis desmarestii*, from the western Mediterranean Spanish marine coast were parasitologically analyzed for the presence of nematodes. All hosts were found parasitized with *Contracaecum* specimens (*n* = 1,517). A representative subsample was genetically identified as *C. rudolphii* sp. A by sequence analysis of the mtDNA *cox*2 gene and the ITS1 and ITS2 regions of the rDNA. This represents the first report of *C. rudolphii* sp. A from the Spanish Mediterranean waters. Population genetic analysis was performed including other *C. rudolphii* sp. A specimens from the west Sardinian and the Tyrrhenian Sea. At the intraspecific level, a significant genetic differentiation (*Fst* ≈ 0.08, *p* < 0.00001) between the metapopulation from the Spanish Mediterranean coast and that from the Sardinian waters was observed; whereas, no differentiation was found between metapopulations of the parasite from the Spanish and the Tyrrhenian Italian coast. The findings highly support the hypothesis of the adaptation of the life cycle of *C. rudolphii* sp. A in brackish and marine ecosystems. Furthermore, the results on the population genetics of *C. rudolphii* sp. A suggest the possible role of the migration routes of wintering populations of cormorants in the Mediterranean Sea in influencing the parasite genetic structure.

## 1. Introduction

The genus *Contracaecum* Raillet and Henry, 1912 includes species maturing in pinnipeds and waterbirds worldwide, representing the most diverse group of the family Anisakidae (1, 2). Considering only the species of the genus maturing in fish-eating birds, 19 species have been so far described as infecting cormorants and pelicans (1, 3–10). Moreover, several morphospecies of the genus have been demonstrated to include sibling species (1, 2, 8, 11–13).

Among them, the *Contracaecum rudolphii* (s.l.) species complex has its most habitual definitive hosts in cormorants belonging to the genus *Phalacrocorax* (Family Phalacrocoracidae) (4, 7, 8, 12, 14, 15). The two cryptic species of the complex parasitizing cormorants belonging to the Family Phalacrocoracidae identified in Europe are *C. rudolphii* sp. A and *C. rudolphii* sp. B (7, 15–17). They are mainly parasites in the Great Cormorant, *Ph. carbo sinensis*. Regarding other sibling species so far included in the *C. rudolphii* (s.l.) complex, *C. rudolphii* sp. C has been detected in *Ph. auritus* from Florida (3). In Australia, the two cryptic species, i.e., *C. rudolphii* sp. D and *C. rudolphii* sp. E, have been found as adult parasites in *Ph. carbo* and *Ph. varius* (8). Moreover, another species indicated as *C. rudolphii* sp. F has been identified as parasitizing the Great Pelican, *Pelecanus occidentalis*, from the Gulf of Mexico (4). Other *Contracaecum* species identified in cormorants are as follows: *C. microcephalum* detected in *Ph. melanoleucos* and *Ph. pygmaeus* from Australia and Montenegro, respectively (3, 9); *C. chubutensis* described in *Ph. atriceps* from the north Patagonian coast (5); *C. australe* discovered in *Ph. brasilianus* from Chile and Argentina (18); and *C. jorgei* observed in *Nannopterum brasilianus* from Argentina (10).

The European Shag, *Phalacrocorax aristotelis*, is a cormorant species commonly found along European marine coasts, mainly observed on rocky seasides and islands. Its nesting habitat in Spain includes a wide range of inaccessible sites on the coast, such as cavities, fissures, and cliff ledges. It is generally sedentary, being the individuals faithful to their first breeding area (19, 20). It comprises three subspecies: *Ph. aristotelis aristotelis*, which inhabits the north-east Atlantic coast, from Iceland to the Iberian Peninsula, including Norway and the Kola Peninsula in Russia; *Ph. aristotelis riggenbachi*, which breeds in the Atlantic coast of Morocco; and finally, *Ph. aristotelis desmarestii*, which is endemic of the Mediterranean and the Black Sea (21). Its diet includes a wide range of fish species, mainly demersal and/or benthic, and could be influenced by their habitat characteristics or the stage of their breeding cycle (22). Studies regarding nematodes' presence in the European Shag are scarce. In Spain, a previous analysis (23) reported the presence of *C. rudolphii* sp. A and *C. septentrionale* in *Ph. a. aristotelis* hosts from the north-west Atlantic coast (Galician coast). In the Mediterranean Sea, *C. rudolphii* sp. A has been identified in *Ph. aristotelis* from the east coast of Sardinia (24), while *C. septentrionale* has been reported in the same hosts from Iceland (25, 26).

The aim of the present study was to (1) identify genetically nematodes of the genus *Contracaecum* parasitizing the European Shag, *Ph. aristotelis desmarestii* from the north-western Mediterranean Sea (coast of Spain); and (2) investigate the population genetic structure of the parasite species in the western Mediterranean Sea areas.

## 2. Materials and methods

### 2.1. Sampling and parasite collection

**Figure 1:**
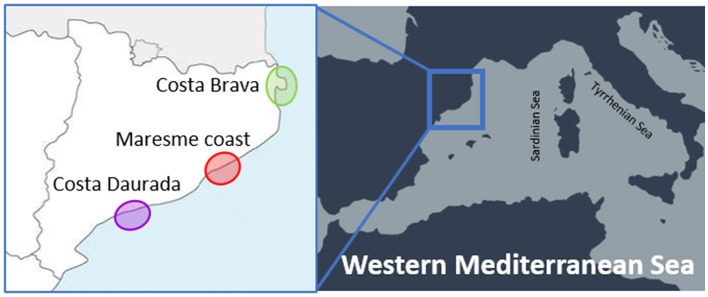
Sampling localities of cormorants from the western Mediterranean Spanish coast (Catalan region).

A parasitological survey was performed on 20 individuals of *Ph. aristotelis desmarestii* collected during the years 2019–2021 from the Catalan-Spanish waters, in the north-western Mediterranean Sea. The collecting sites of the cormorants are grouped in three different areas, corresponding to the northern (Costa Brava), the central (Maresme coast), and the southern (Costa Daurada) regions of the Catalan coast, as shown in Figure 1. Cormorants were found dead and subsequently frozen until their parasitological analysis. Stomach dissection was carried out at the laboratory for the presence of larval and/or adult nematodes. All the recovered specimens were washed in saline solution, morphologically referred following Berland (27) and Hartwich (14) criteria, and subsequently fixed in 70% ethanol.

### 2.2. Genetic-molecular analysis

Identification at the species level of the recovered nematodes was performed on a subsample of 75 specimens of *Contracaecum*, 28 from the Costa Brava, 26 from the Costa Daurada, and 21 from the Maresme coast. A minimum of at least two specimens from each cormorant individual was selected for DNA extraction, which was performed using the Quick-DNA Midiprep Plus Kit (Zymo Research, Irvine, USA). Direct sequence analysis of the two genes, i.e., the mtDNA *cox*2 and the ITS region of the rDNA, was carried out on each nematode specimen. The mitochondrial *cox*2 gene was amplified using 211F and 210R primers, with a hybridization temperature of 46°C (13, 17). ITS region of the rDNA was amplified using NC5 and NC2 primers at 55°C (28). For both regions, the PCR products were sequenced by Bio-Fab Research (Roma, Italy) using the same primers.

### 2.3. Sequences analysis

Sequences were aligned using the Clustal X software and adjusted manually (29). Phylogenetic analysis of mtDNA *cox*2 and ITS rDNA regions of the present study, along with other *Contracaecum* species previously deposited in GenBank, was carried out by Bayesian analysis (BI) using MrBayes software (30). The sequences used for the analysis are reported in Table 1. Because the entire ITS sequence was not available in GenBank, ITS1 and ITS2 sequences were used to carry out the concatenated sequence analysis using Sequence Matrix software (36). The best-fit substitution models were determined by JModeltest software using the Akaike Information Criterion (AIC) (37). The Bayesian posterior probability analysis was carried out with 1,000,000 generations and a subsampling frequency of 100. Posterior probabilities were estimated and used to assess support for each branch, considering a well-supported value of 0.90.

**Table 1:** List of mtDNA *cox*2 and ITS rDNA sequences used for the Bayesian inference phylogenetic analysis.

**Nematode species**	**Accession number**	**DNA region**	**Reference**
*C. rudolphii* sp. A	OP690535/40	mtDNA *cox*2	Present study
*C. rudolphii* sp. A	EF513502	mtDNA *cox*2	(31)
*C. rudolphii* sp. A	EF558891	mtDNA *cox*2	(31)
*C. rudolphii* sp. B	EF558893/94	mtDNA *cox*2	(31)
*C. rudolphii* sp. B	MK496485/86/87	mtDNA *cox*2	(7)
*C. rudolphii* sp. F	JF727879	mtDNA *cox*2	(4)
*C. septentrionale*	EF122205	mtDNA *cox*2	(31)
*C. septentrionale*	EF513512	mtDNA *cox*2	(31)
*C. australe*	GQ847532/33	mtDNA *cox*2	(18)
*C. bioccai*	EF513494/95	mtDNA *cox*2	(31)
*C. overstreettii*	EU852343/44	mtDNA *cox*2	(6)
*C. gibsoni*	EU852337/38	mtDNA *cox*2	(6)
*C. micropapillatum*	EF122206	mtDNA *cox*2	(31)
*C. micropapillatum*	EF513514	mtDNA *cox*2	(31)
*C. microcephalum*	EF513517/18	mtDNA *cox*2	(31)
*P. ceticola*	DQ116435	mtDNA *cox*2	(32)
*C. rudolphii* sp. A	OP677791	ITS1 rDNA	Present study
*C. rudolphii* sp. A	OP677785	ITS2 rDNA	Present study
*C. rudolphii* sp. A	EU678869	ITS rDNA	(24)
*C. rudolphii* sp. C	FJ822037	ITS rDNA	Unpublished
*C. rudolphii* sp. D	FM210251/52/56	ITS1 rDNA	(8)
*C. rudolphii* sp. D	FM210261/62/63	ITS2 rDNA	(8)
*C. rudolphii* sp. E	FM210257/58/60	ITS1 rDNA	(8)
*C. rudolphii* sp. E	FM210269/70/71	ITS2 rDNA	(8)
*C. rudolphii* sp. F	JF424597	ITS rDNA	(4)
*C. rudolphii* sp. B	MK496497/98	ITS1 rDNA	(7)
*C. rudolphii* sp. B	MT096419/20	ITS2 rDNA	(7)
*C. australe*	HQ389545	ITS1 rDNA	(18)
*C. australe*	HQ389547	ITS2 rDNA	(18)
*C. chubutensis*	HQ389546	ITS1 rDNA	(18)
*C. chubutensis*	HQ389548	ITS2 rDNA	(18)
*C. bioccai*	JF424598	ITS rDNA	(7)
*C. septentrionale*	AJ634784	ITS1 rDNA	(12)
*C. septentrionale*	AJ634787	ITS2 rDNA	(12)
*C. microcephalum*	FM177524/26	ITS1 rDNA	(9)
*C. microcephalum*	FM177527/29	ITS2 rDNA	(9)
*C. bancrofti*	EU839566/73	ITS1 rDNA	(9)
*C. bancrofti*	FM177883/87	ITS2 rDNA	(9)
*C. multipapillatum* sp. D	AM940056/59	ITS1 rDNA	(33)
*C. multipapillatum* sp. D	AM940060/61	ITS2 rDNA	(33)
*C. pyripapillatum*	AM940062/63	ITS1 rDNA	(33)
*C. pyripapillatum*	AM940066/67	ITS2 rDNA	(33)
*C. multipapillatum* sp. E	OL830809	ITS rDNA	(34)
*C. multipapillatum* sp. E	OL830790	ITS rDNA	(34)
*A. suum*	MH030604	ITS rDNA	(35)

### 2.4. Intraspecific genetic diversity

Intraspecific genetic diversity of *C. rudolphii* sp. A population from the Spanish Mediterranean coast identified in the present study, inferred from the mtDNA *cox*2 sequences analysis, was compared with other conspecific populations previously analyzed by the same genetic markers (7) from cormorants (*Ph. carbo sinensis*) of several marine localities of the Tyrrhenian coast of Italy. In particular, they were from the Orbetello lagoon, the estuary of the Fiora and Marta rivers, and the Tarquinia salterns. In addition, sequences of the mtDNA *cox*2 of the same parasite species, available in GenBank, reported from *Ph. c. sinensis* of the western Sardinian coast [see Amor et al. (38)] were also considered. Population genetic analysis was performed by using DnaSP V5.10.01 (39), estimating the genetic diversity on the following standard statistical parameters: number of haplotypes (*Nh*), number of private haplotypes (*Nuh*) nucleotide diversity (π), haplotype diversity (*Hd*), average number of differences (*K*), and number of polymorphic sites (*S*). Spatial analysis of molecular variance (AMOVA) and analysis of the estimation of *Fst* values were conducted on the genetic datasets inferred from the populations of *C. rudolphii* sp. A from the distinct sampling areas with ARLEQUIN version 3.5 with 1,000 permutations, establishing the level of significance to a *P* < 0.05 (40, 41). A haplotype parsimony network was constructed using PopART (42). The analysis was performed using the statistical parsimony procedure (95% parsimony connection limit), implemented in TCS (43). Circle sizes are proportional to the corresponding haplotype frequencies.

## 3. Results

The parasitological analysis of the *Ph. aristotelis desmarestii* individuals allowed the identification of 1,517 nematode specimens which were morphologically referred to as third-stage larvae (L3), preadults, and adults of *C. rudolphii* (s.l.). All cormorants were found to be parasitized with nematode specimens (Prevalence, P = 100%), being the parasites found in the stomach and along the esophagus of the hosts.

**Figure 2:**
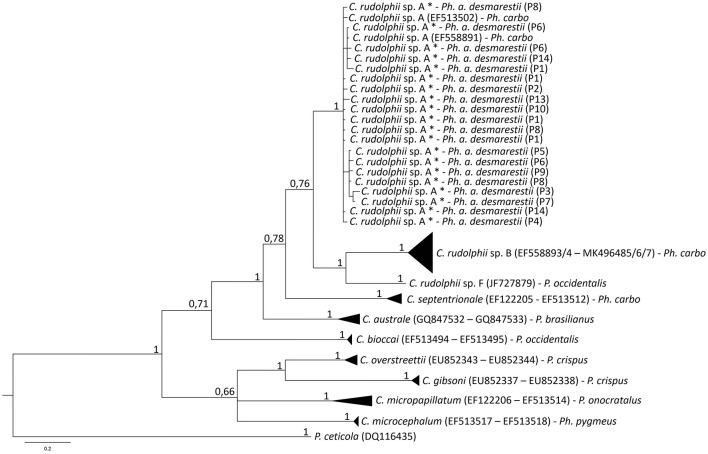
Bayesian inference phylogenetic tree based on sequences of mtDNA *cox*2 gene of specimens of *C. rudolphii* sp. A obtained in the present study with respect to the other *Contracaecum* spp. sequenced at the same gene locus previously deposited in GenBank. Definitive host species of each *Contracaecum* species sequence are indicated. Specimens obtained in the present study are marked with an asterisk including in brackets the definitive host specimen code. The analysis was performed by MrBayes software (30) using the GTR+I+G (I+G = 0.5482) substitution model as determined by JModeltest software (37). *P. ceticola* was used as an outgroup.

**Figure 3:**
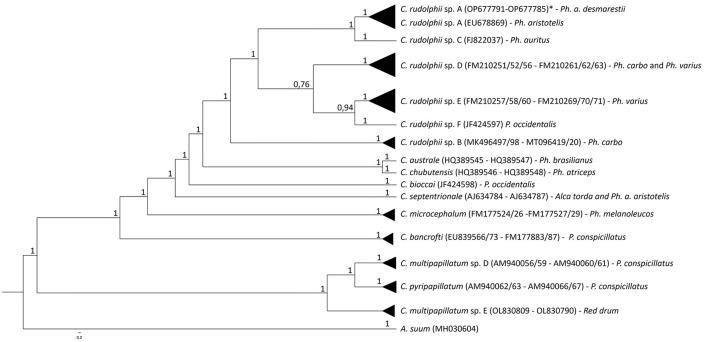
Bayesian inference phylogenetic tree based on sequences of ITS1 and ITS2 rDNA regions of specimens of *C. rudolphii* sp. A obtained in the present study with respect to the other *Contracaecum* spp. sequenced at the same locus previously deposited in GenBank. Definitive host species of each *Contracaecum* species sequence are indicated. The analysis was performed by MrBayes software (30) using TVM+G (G = 0.3353) substitution model as determined by JModeltest software (37). *A. suum* was used as an outgroup.

Considering the obtained sequences, the best-fit substitution models were GTR+I+G (I+G = 0.5482) and TVM+G (G = 0.3353) for mtDNA *cox*2 and ITS rDNA regions, respectively. The mtDNA *cox*2 and the ITS1 and ITS2 rDNA sequences analysis of the *C. rudolphii* (s.l.) specimens allowed us to identify all the nematode specimens as *C. rudolphii* sp. A, including 33 adults, 21 preadults, and 21 larvae. DNA sequences from mtDNA *cox*2 and ITS1 and ITS2 rDNA regions were deposited in GenBank under the accession numbers OP690535-40 (mtDNA *cox*2), OP677791-96 (ITS1 region of rDNA), and OP677785-90 (ITS2 region of rDNA). Indeed, the Bayesian inference (BI) phylogenetic tree, based on mtDNA *cox*2 sequences (Figure 2), indicated that all the *C. rudolphii* sp. A sequences here obtained from specimens parasitizing *Ph. a. desmarestii*, clustered together, with high probability values in a well-supported clade, with other *C. rudolphii* sp. A sequences previously deposited in GenBank from specimens parasitizing *Ph. carbo sinensis* (accession numbers EF513502 and EF558891). This clade was clearly distinct from the other two clades obtained for the other sibling species, *C. rudolphii* sp. B and *C. rudolphii* sp. F, reported in *Ph. carbo* and *P. occidentalis*, respectively. Similarly, sequences of the other *Contracaecum* species included in the tree, parasitizing fish-eating bird hosts of the genera *Phalacrocorax* and *Pelicanus*, were grouped into distinct and well-supported clades (Figure 2). In the same vein, the BI phylogenetic tree based on rDNA ITS1 and ITS2 sequences (Figure 3) indicated that all the sequences obtained from *Contracaecum* spp., previously sequenced at the mtDNA *cox*2 locus, were grouped in a well-supported clade with a reference sequence of *C. rudolphii* sp. A from the same host (*Ph. aristotelis*) (accession number EU678869). This clade was phylogenetically distinct in comparison with the other six clades obtained for each sibling species, i.e., *C. rudolphii* sp. B, *C. rudolphii* sp. C, *C. rudolphii* sp. D, *C. rudolphii* sp. E, and *C. rudolphii* sp. F, as well as for the other *Contracaecum* species, from hosts of the *Phalacrocorax* and the *Pelicanus* genera (Figure 3).

**Figure 4:**
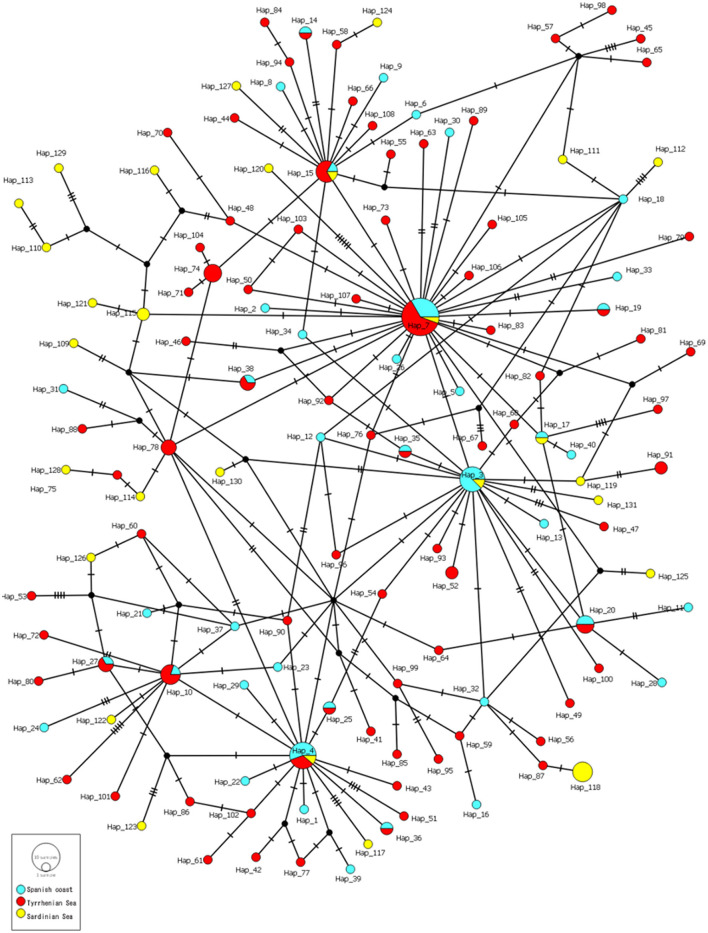
TCS network of *C. rudolphii* sp. A sequences of mtDNA *cox*2 dataset from *Ph. a. desmarestii* obtained in the present study and *Ph. c. sinensis* from Mattiucci et al. (7) and Amor et al. (38). Hatch marks indicate mutations. Circle sizes are proportional to the number of shared individuals per haplotype.

The hierarchical gene diversity analysis by AMOVA indicated that 3.69% of the genetic variation of *C. rudolphii* sp. A from the western Mediterranean localities here considered was expressed among populations while 96.31% was observed within populations. Because no significant intraspecific genetic variation was found among specimens from the Catalan-Spanish coast analyzed in the present work, they were considered as a single population. Estimates of genetic differentiation among *C. rudolphii* sp. A population of the three analyzed areas from the west Mediterranean, i.e., the Spanish Catalan, the Tyrrhenian, and the Sardinian coasts, inferred from the *Fst* value and their *P*-values, are shown in Table 2. Significant genetic differentiation was found among *C. rudolphii* sp. A metapopulations from the Sardinian coast compared to those from the Spanish region examined here (*Fst* ≈ 0.08, *p* < 0.00001). Similarly, significant *Fst* values were found between *C. rudolphii* sp. A populations from the Sardinian and Tyrrhenian coasts (*Fst* ≈ 0.06, *p* < 0.00001). In contrast, no significant genetic differentiation was observed between the populations from the Mediterranean Spanish coast (Catalan region) and the Tyrrhenian one (*Fst* ≈ 0.005, *p* > 0.05). The haplotype parsimony network (TCS) analysis, inferred from *cox*2 mtDNA of *C. rudolphii* sp. A, revealed a star-like tree as shown in Figure 4. In the Spanish coast population of the parasite species, 40 haplotypes were detected, while 80 and 28 haplotypes were identified from the Tyrrhenian and Sardinian populations, respectively (Table 3). The Hap7 was the most frequent haplotype shared by all three metapopulations. Hap4 and Hap15 were also frequently observed, shared by several individuals from all geographical areas. Whereas, the Hap3 was mainly shared by individuals from the Spanish coast. However, in these four major haplotypes, the relative frequency of individuals from the Sardinian waters was very low, comprising mainly Spanish and Tyrrhenian individuals. In addition, specimens from the Sardinian coast presented a high frequency of unique haplotypes. As shown in Table 3, the metapopulation from the Sardinian area showed the highest values of nucleotide diversity (per site) (π = 0.112) and the average number of nucleotide differences (*K* = 5.807). The haplotype diversity (*Hd*) was high in all three populations, with values ranging between 0.969 and 0.986.

**Table 2:** Population pairwise *Fst* estimates inferred from mtDNA *cox*2 gene sequences analysis among *C. rudolphii* sp. A from the different western Mediterranean marine waters studied.

	**Spanish Catalan coast**	**Tyrrhenian Sea coast**	**Western Sardinian coast**
**Spanish Catalan coast**	-	0.1475 ± 0.0117	0.0000 ± 0.0000
**Tyrrhenian Sea coast**	0.00467	-	0.0000 ± 0.0000
**Western Sardinian coast**	0.07797	0.06298	-

Below the diagonal: conventional *Fst* from haplotype frequencies; above the diagonal: P-values of *Fst*.

**Table 3:** Genetic diversity of *C. rudolphii* sp. A, inferred from the mtDNA *cox*2 gene, from the cormorants' definitive host, of the different western Mediterranean marine waters studied.

**Population**	***N***	***Nh***	***Nuh***	**π ±*SD***	***Hd* ±*SD***	***K***	***S***
Spanish Catalan coast	56	40	26	0.00681 ± 0.00053	0.969 ± 0.013	3.425	40
Tyrrhenian Sea coast	110	80	68	0.00871 ± 0.00050	0.986 ± 0.005	4.379	87
Western Sardinian coast	33	28	23	0.01174 ± 0.00098	0.979 ± 0.018	5.807	44

*N*, Number of sequences analyzed; *Nh*, number of haplotypes; *Nuh*, Number of private haplotypes; π, nucleotide diversity (per site); *Hd*, haplotype diversity; *SD*, standard deviation; *K*, average number of nucleotide differences; *S*, number of polymorphic sites.

## 4. Discussion

Several *Contracaecum* species included in the *C. rudolphii* (s.l.) complex have been identified as parasitizing cormorants, worldwide. In the present study, we report the first genetic identification of *C. rudolphii* sp. A in the European Shag, *Ph. aristotelis desmarestii*, from the western Mediterranean Spanish coasts.

The multi-marker genetic approach using the mtDNA *cox*2 gene and the ITS rDNA region allowed the specific identification of all the nematode samples studied. The genetic identification of the sibling species of the *C. rudolphii* (s.l.) complex is important in order to understand their ecology and geographical distribution, but also the phylogenetic relationship among them. Both BI phylogenetic trees, based on the mtDNA *cox*2 gene locus and the ITS1 and ITS2 of the rDNA region, respectively, have shown that the *C. rudolphii* sp. A specimens were genetically distinct from the other species of the *C. rudolphii* (s.l.) complex, including sequences of *C. rudolphii* sp. B, *C. rudolphii* sp. C, *C. rudolphii* sp. D, *C. rudolphii* sp. E, and *C. rudolphii* sp. F (3, 4, 8, 13, 44). As shown in Figure 3, and in accordance with previous studies, a closer genetic relationship was observed between *C. rudolphii* sp. A with the other sibling species rather than with *C. rudolphii* sp. B. (7, 9). In this sense, D'Amelio et al. (3), when analyzing the phylogenetic tree inferred from the *rrnS* rDNA region, found the closest relationship between *C. rudolphii* sp. A and *C. rudolphii* sp. C, rather than between *C. rudolphii* sp. A and *C. rudolphii* sp. B. Although heterozygote genotypes between *C. rudolphii* sp. A and C *rudolphii* sp. B have been reported, inferred from the ITS rDNA region (38, 45), their genetic analysis should be assessed by using a multi-nuclear diagnostic loci approach, as shown between sibling species of other anisakid nematodes, i.e., the species of the *Anisakis simplex* (s.l.) complex (46–49). Discovering novel nuclear diagnostic markers between the two species would also improve the investigation of the possible existence of hybridization/introgression events between them.

Cormorants of the *Ph. a. desmarestii* subspecies are sedentary animals that habit strictly marine ecosystems and are endemic to the Mediterranean Sea (21). Data regarding the nematode presence in this host are scarce. In the Mediterranean waters, *C. rudolphii* sp. A has been identified parasitizing European Shag specimens on the Sardinian coasts (24). In the north-western Spanish waters (Atlantic Galician coast), the presence of *C. rudolphii* sp. A was reported at high prevalence values parasitizing cormorants of the subspecies *Ph. aristotelis aristotelis* (23). Those records, combined with the present finding of *C. rudolphii* sp. A in *Ph. aristotelis desmarestii* from the western Mediterranean Spanish coast and the absence of the sibling species *C. rudolphii* sp. B, confirm the adaptation of the former anisakid species to marine and brackish-water ecosystems, as suggested by Mattiucci et al. (7, 15). This hypothesis was supported by the high relative proportion of *C. rudolphii* sp. A found in *Ph. carbo sinensis* habiting in brackish environments of Italy, whereas the sibling species *C. rudolphii* sp. B infecting the same cormorant species was mainly found living in freshwater environments, such as rivers and lakes of Italy (7). Similar proportions of infection, with the species *C. rudolphii* sp. A from brackish cormorants (38) and with *C. rudolphii* sp. B in cormorants from freshwater environments (50) have been reported later. It has been hypothesized that the observed differential spatial distribution of *C. rudolphii* sp. A and *C. rudolphii* sp. B in distinct aquatic ecosystems could also be related to the differential feeding ecology and the behavior of distinct wintering populations of *Ph. carbo sinensis* in European areas (7). In this sense, the sedentary pattern and the feeding behavior of the *Ph. a. desmarestii*, the definitive host herein studied, would be responsible for the maintenance of the life cycle of *C. rudolphii* sp. A along the Catalan marine coastal ecosystem. The low mobility of the *Ph. a. desmarestii* could also help to maintain the genetic differentiation of the *C. rudolphii* sp. A population observed in the present study, justifying the relatively high number of private haplotypes (*n* = 26) shown by the parasite species in this area (Figure 4). On the other hand, the migration pattern of the Great Cormorant, *Ph. carbo*, which acts as the main definitive host of *C. rudolphii* sp. A in European waters might explain the population genetic differentiation here found between the metapopulation of *C. rudolphii* sp. A from Sardinia in comparison with those conspecific ones from the Catalan and Tyrrhenian areas (Table 2). This fish-eating bird presents its high migration pattern along the brackish and freshwater environments of Europe, inhabiting, among the others, the Catalan and the Tyrrhenian Mediterranean waters during its non-breeding season (51, 52). Although the low number of samples from the Sardinian Sea herein studied (*n* = 33) should be considered, the existence of a population of *Ph. carbo* living during the winter season in Sardinia, which could have a different breeding area in comparison with those populations of *Ph. carbo* wintering in the Spanish and Tyrrhenian areas, might explain the parasite genetic differentiation observed in the present study. In support of this hypothesis, a higher presence through Sardinia has been observed in some *Ph. carbo* populations during its migration routes (53). Moreover, Marion and Gentil (54) detected the presence of two genetically distinct cormorant populations of *Ph. carbo*, one along the Sardinia coast and the other one along the Italic Peninsula, as inferred from the mtDNA *cox*2 gene, which could also explain the present finding of a certain genetic sub-structuring of *C. rudolphii* sp. A, harbored by cormorants analyzed from those areas, i.e., Sardinia (38) and Latium region (7). Indeed, *C. rudolphii* sp. A from cormorants of the Sardinian coast also showed a high number of private haplotypes (*n* = 23), not shared with the other populations, despite the lower number of specimens analyzed, which is in accordance with the estimation of *Fst* values herein obtained. In addition, the most frequent haplotypes observed in Figure 4 seemed to be shared mainly by specimens from the Spanish and the Tyrrhenian coasts. The influence of the population structure and the migration route of the definitive hosts in shaping the genetic structure of anisakid nematodes has also been observed in *Anisakis* spp. (55) and *Uncinaria lucasi* (56). In this sense, it is suggested that the interaction among definitive, intermediate, and paratenic hosts influences the genetic structure of parasite populations, being the geographic origin of the host species a major driver (55). However, the analysis of a high number of specimens of *C. rudolphii* sp. A from cormorants collected from different brackish and marine ecosystems of European countries, as well as knowledge on the migration patterns of different populations of its phalacrocoracid definitive host, is necessary in order to shed light on the population genetic structure of this waterbird anisakid species.

The high prevalence of *C. rudolphii* sp. A (P = 100%) observed in the present study from the analyzed cormorant specimens is in accordance with that previously reported by Abollo et al. (23) in *Ph. a. aristotelis* from the Spanish Atlantic coast and with those recorded in *Ph. c. sinensis* from the Tyrrhenian Sea (7, 38). Comparison with *C. rudolphii* sp. A from the population of the European Shag of Sardinia is not possible because of the lack of epidemiological data (24). However, despite the high prevalence values herein observed, the life cycle of *C. rudolphii* sp. A in the Catalan Spanish coasts is not clear. Various studies analyzing several intermediate/paratenic hosts from this area, such as *Micromesistius poutassou* (57)*, Sardina pilchardus* (58), *Phycis blennoides* (59), and *Merluccius merluccius* (60), among others, have not detected *Contracaecum* spp. larvae in these fish. Nevertheless, along the Spanish Mediterranean coast, some studies have identified *Contracaecum* spp. larvae parasitizing other fish species, including *Anguilla anguilla* (61), *Scomber scombrus, Trachurus trachurus* (62), *Pagellus erythrinus, Trisopterus minutus*, and *Engraulis encrasicolus* (59). Still, specimens were identified only at the genus level, using morphological criteria and without performing the molecular identification of the parasites. In other areas of the Mediterranean, such as Sardinian waters and the Tyrrhenian Sea, specific identification of *C. rudolphii* sp. A parasitizing various intermediate/paratenic hosts, including *Sparus aurata* (63), *Trachurus* sp., *Mullus* sp., *Illex coindettii* (64), *A. anguilla, Dicentrarchus labrax* (7, 65), *Gobius niger, G. paganellus*, and *Solea solea* (65) has been reported. Studies regarding the genetic identification of *Contracaecum* larvae in fish species, likely included in the prey items list of the *Ph. a. desmarestii*, from the western Mediterranean Spanish coast, should be accomplished for a better understanding of the *C. rudolphii* sp. A life cycle in the studied area.

## Data availability statement

The datasets presented in this study can be found in online repositories. The names of the repository/repositories and accession number(s) can be found in the article/ supplementary material.

## Ethics statement

Ethical review and approval was not required for the animal study because the cormorants used in the present study were found dead and no permit and/or ethical consideration were needed.

## Author contributions

MC, CT, and JB performed a cormorant necropsy and the collection and morphological identification of parasites. XR-G, MP, and SM contributed to the design of the study, performed molecular and phylogenetic analyses of selected parasites, and wrote the manuscript. All authors contributed to the manuscript revision, and read and approved the submitted version.
